# Plastic Surgery Complications: A Review for Emergency Clinicians

**DOI:** 10.5811/westjem.2020.6.46415

**Published:** 2020-09-25

**Authors:** Tim Montrief, Kasha Bornstein, Mark Ramzy, Alex Koyfman, Brit J. Long

**Affiliations:** * University of Miami Miller School of Medicine, Department of Emergency Medicine, Miami, Florida; † University of Miami Miller School of Medicine, Miami, Florida; ‡ Maimonides Medical Center, Department of Emergency Medicine, Brooklyn, New York; § The University of Texas Southwestern Medical Center, Department of Emergency Medicine, Dallas, Texas; ¶ Brooke Army Medical Center, Department of Emergency Medicine, Fort Sam Houston, Texas

## Abstract

The number of aesthetic surgical procedures performed in the United States is increasing rapidly. Over 1.5 million surgical procedures and over three million nonsurgical procedures were performed in 2015 alone. Of these, the most common procedures included surgeries of the breast and abdominal wall, specifically implants, liposuction, and subcutaneous injections. Emergency clinicians may be tasked with the management of postoperative complications of cosmetic surgeries including postoperative infections, thromboembolic events, skin necrosis, hemorrhage, pulmonary edema, fat embolism syndrome, bowel cavity perforation, intra-abdominal injury, local seroma formation, and local anesthetic systemic toxicity. This review provides several guiding principles for management of acute complications. Understanding these complications and approach to their management is essential to optimizing patient care.

## INTRODUCTION

**Figure 1:**
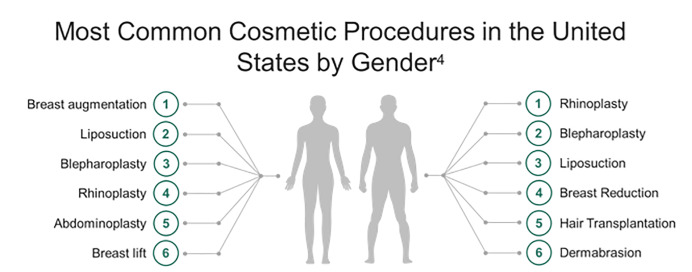
Most common cosmetic procedures in the United States in 2017 by gender. Statistics available at https://www.plasticsurgery.org/documents/News/Statistics/2017/plastic-surgery-statistics-full-report-2017.pdf.

The number of aesthetic surgical procedures performed in the United States is increasing rapidly. Over 1.5 million aesthetic surgical procedures were performed in 2015.^1^ Breast augmentation and suction-assisted lipectomy (SAL), also known as liposuction, are the most frequently performed cosmetic procedures in the US with over 600,000 performed annually (Figure 1).^2^–^4^ Cosmetic procedures are lucrative, and in the absence of legal restrictions, are increasingly being performed in outpatient settings by non-plastic surgeons and even non-physicians.^5^,^6^ Growing medical tourism has spurred demand for cosmetic surgery in Europe, South America, and Southeast Asia.^6^–^8^ A survey distributed to 2000 active members of the American Society of Plastic Surgeons (ASPS) showed that 51.6% of respondents noted an increasing trend in the number of patients presenting with complications from surgical tourism.^9^ Public perception of these surgeries as minor procedures contributes to risks for major complications with potentially fatal consequences, with reported mortality of 1 per 5000 procedures.^5^,^10^–^12^ Emergency clinicians should be aware of possible complications.

## METHODS

This review focuses on the complications of the most common surgical procedures including liposuction, breast augmentation, abdominoplasty, and subcutaneous injections. We describe the expected presentations, evaluation, and emergent care required to manage post-cosmetic surgery complications. We performed a literature search of Medline, PubMed, and Google Scholar for “plastic surgery,” “complication,” “liposuction,” “mammoplasty,” “abdominoplasty,” “surgical site infection,” “dehiscence,” “fat embolism,” “perforation,” “local anesthetic systemic toxicity.” The database search was conducted from inception of each database to April 1, 2020. We evaluated case reports and series, retrospective and prospective studies, systematic reviews and meta-analyses, and other narrative reviews. We also reviewed guidelines and supporting citations of included articles. The literature search was restricted to studies published in English, with focus on emergency medicine (EM) and critical care literature.

## RESULTS

We decided by consensus which studies to include for the review. When available, systematic reviews and meta-analyses were preferentially selected. These were followed sequentially by randomized controlled trials, prospective studies, retrospective studies, case reports, and other narrative reviews when alternate data were not available. There is a notable absence regarding the discussion of plastic surgery complications in the EM and critical care literature. A total of 114 resources were used for the construction of this narrative review.

## DISCUSSION

### Brief Review of Surgical Techniques

#### Liposuction

Emerging in the 1970s, SAL is one of the most widespread aesthetic surgeries practiced.^13^ Outpatient SAL is typically performed under local anesthesia and is used commonly on the buttocks, back, thighs, face, chest, and abdomen. The predominant technique, *microcannula tumescent liposuction*, consists of suction removal of fat from deep subcutaneous layers via aspiration cannulae introduced through small skin incisions.^14^ Several liters of tumescent solution consisting of dilute local anesthetic, epinephrine, and saline are infiltrated into the subcutaneous tissue, percolating through tissue layers prior to aspiration.^15^ The saline balloons tissues (tumescence), epinephrine causes vasoconstriction which decreases bleeding, and lidocaine induces local anesthesia.^16^ Generally, incisions are left open to drain remaining fluid.^17^ Duration of SAL procedures is typically 3–4 hours. The volume of subcutaneous fat that can be extracted is approximately 4–5 liters.^17^

#### Mammoplasty

Mammoplasty, including breast reduction and augmentation, is a common aesthetic surgical procedure. Mammoplasty typically requires inpatient admission, especially if combined with another procedure such as abdominoplasty.^18^ Many surgical techniques exist for breast augmentation. All involve incisions extending caudally between breast and subcutaneous tissue, exposing the pectoral fascia. A rent is then made in the fascia, and fibers of the pectoralis major are split, forming a submuscular pocket into which breast prostheses are placed.^19^ Surgical techniques and implant technology evolved over the course of the 20^th^ century. Due to capsular contracture with older prostheses, manufacturers began to design round, smooth-surfaced implants that can move within surgical pockets.^20^ Implantation of synthetic and biological matrices such as acellular dermal matrix in surgical breast reconstruction is becoming increasingly common.^21^ Implant-based breast reconstruction includes one- or two-stage procedures where expanders or permanent implants are placed to contour breast appearance, with or without use of reinforcing matrices.^21^ Breast reduction consists of resection of breast tissue, skin, and parenchyma with formation of a free skin flap. Liposuction may be performed beyond the area of skin resection to shape tissue.^22^

#### Abdominoplasty

Abdominoplasty is used to reshape body contours by means of excising redundant skin and fat tissue to remodel the abdominal wall. Contemporary techniques use three main characteristics: abdominal flap dissection, plication of the rectus abdominis fascia, and resection of skin and underlying Scarpa fascia-adjacent subdermal tissue. Abdominoplasty is now preceded by or performed concurrently with liposuction in 90% of cases.^23^ This practice preserves nerve and blood supply to the abdominal skin and minimizes “dead space,” which poses risks for postoperative complications.^24^

#### Subcutaneous Injections

Subcutaneous injections of dermal “fillers” include a variety of substances injected into the body for soft tissue augmentation. One of the most common sites is the buttocks.^25^ Surgical enhancement of buttock volume has been performed for decades, primarily using silicone or autologous fat injection.^26^ The procedural technique for silicone placement is analogous to breast augmentation.

### Complications of Cosmetic Surgical Procedures

**Figure 2:**
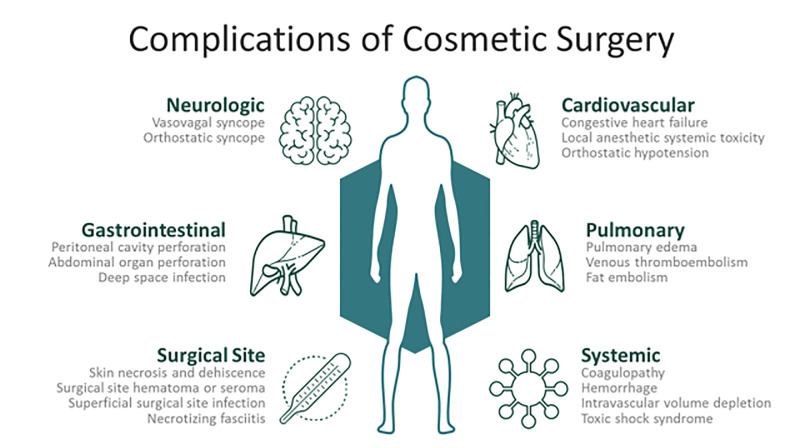
Common postoperative complications of cosmetic surgery.

Physiologic risks of plastic surgery procedures are comparably less than those of other surgical subspecialties. Aesthetic surgical procedures are typically elective and usually performed on an outpatient basis in relatively healthy patient populations. Despite these factors, significant risks exist for postoperative complications. Common complications include infections, local anesthetic systemic toxicity (LAST), electrolyte and hematologic abnormalities, intravascular fluid shifts, and wound complications. Postoperative complications may be immediate, such as LAST, or delayed up to months, as may occur with surgical site hematomas.^2^,^6^ Figure 2 depicts common postoperative complications and clinical findings that may assist in distinguishing etiologies leading to ED presentation.

### Post-surgical Complications: Evaluation and Management

#### Antibiotic Use and Surgical Site Infections (SSI)

The dissected subcutaneous layer created in cosmetic procedures creates an optimal environment for bacterial growth. This presenting risk for infections ranges from cellulitis to life-threatening necrotizing fasciitis (ie, infections invading fascial planes with tissue necrosis). No specific guidelines for perioperative prophylaxis exist for cosmetic surgeries. Prophylactic perioperative antibiotic use is controversial except in breast surgeries, where antibiotic prophylaxis is universally recommended, particularly in surgeries using implants, drains, or mesh.^27^–^30^ Antibiotic prophylaxis should cover both Gram positive and negative bacteria. Of these, the most common culprit for postoperative infection is *Staphylococcus aureus*.^31^ Duration of postoperative antibiotic courses range between 24 hours to 14 days, with oral antibiotics frequently continued until surgical drains are removed.^31^

After local fluid collections, postoperative SSIs are the most common local wound complication. SSIs vary by nature of the procedure performed. Breast surgeries have higher associated incidence of wound complications, including infection.^32^ Postoperative infections are present in up to 35% of breast surgeries. Most literature suggests an overall incidence of less than 1% in all aesthetic surgeries combined.^27^,^33^–^35^ Reported SSI incidence following abdominoplasty is variable, ranging from 0.2% to 32.6% of patients in large series.^36^–^38^ Cárdenas et al reported an SSI incidence of 0.09%, with only one infection in 1047 patients who underwent liposuction.^39^,^40^

The Centers for Disease Control and Prevention (CDC) defines SSI as infections related to an operative procedure occurring at or near surgical incisions within 30 days of the procedure. The CDC categorizes SSI into *superficial* and *deep* presentations.^41^ Superficial SSIs are an infection of the dermis and subcutaneous tissue, presenting similarly to cellulitis with imaging findings of fascial thickening, septation of subcutaneous fat, and/or lymph node enlargement.^42^ Clinical assessment is imperative, as uncomplicated cellulitis may appear similar to normal postoperative tissue on ultrasound and computed tomography (CT).^42^ Symptoms such as fever, local warmth, erythema, and tenderness to palpation should be considered alongside laboratory results when evaluating these patients.^42^ Consultation with the operative surgeon is recommended, as he or she may help facilitate outpatient follow-up and appropriate antibiotic choice based on facility antibiogram. Infectious Diseases Society of America guidelines for moderate, non-purulent skin and soft tissue infections recommend penicillin, ceftriaxone, cefazolin, or clindamycin.^43^ If the patient has had fat grafting with infection of the graft site or harvest site, a 2–3 day admission with intravenous (IV) antibiotics may be necessary for rapidly progressing infection.^42^–^44^ There is growing concern about chronic, refractory inflammation developing after aesthetic surgeries necessitating admission for IV antibiotics.^44^ The etiology underlying these chronic cases is thought to be antibiotic-resistant bacteria and fungi and rapidly growing mycobacteria.^45^,^46^

Deep SSIs involve the deep soft tissue planes and may extend to fascia and visceral organ structures. Postoperative infection in cosmetic surgery patients poses a diagnostic challenge as edema, color changes, and blistering can result from the initial procedure, thus concealing infectious processes.^47^ Constitutional signs and symptoms of infection, including fever, chills, and rigors, should raise suspicion for development of SSI and/or associated sepsis.^43^,^44^,^47^ Deep infections may also evolve into necrotizing fasciitis, which has been described after cosmetic surgeries, most frequently SAL.^48^–^50^ Necrotizing fasciitis is a surgical emergency necessitating prompt antibiotic treatment, early surgical consultation, and often radical debridement of necrotic tissue.^51^ CT with IV contrast is the most sensitive modality for diagnosing necrotizing fasciitis and evaluating the extent of disease. While radiographic findings parallel those of cellulitis, necrotizing fasciitis may be distinguished by gas in the muscle layer.^42^,^52^

Bacteria are the most common causative agents underlying postoperative SSI. *S. aureus, S. epidermidis*, Streptococci A and B, *Streptococcus pyogenes*, *Klebsiella pneumoniae, Bacillus*, and *Propionibacterium* are most often implicated. Corynebacterium, *Pseudomonas aeruginosa, Escherichia coli*, and *Enterobacteriaceae* are also occasionally implicated.^53^–^55^

Infection remains the greatest risk of implant-based breast reconstruction, particularly in the setting of mesh implantation. Prosthesis infections can lead to complications ranging from mild SSIs, including superficial cellulitis, to surgical revision for chronic wounds, implant failure, and life-threatening sepsis.^56^ In the setting of breast augmentation with mesh use, infection may lead to bacterial biofilm development with subsequent capsular contracture and rib osteomyelitis.^57^–^61^ Approximately two-thirds of postoperative breast infections develop within one month. One report noted 13.3% of patients developed infections three months after surgery, 8.3% after more than six months, and sporadically up to decades following surgery.^57^ Risk factors for development of an SSI after breast surgery include older age, female gender, elevated body mass index (BMI), current tobacco smoking, diabetes mellitus, immunosuppressed states, multiple concurrent procedures, and undergoing procedures elsewhere besides the breast or face.^40^

ED management of suspected deep SSI includes early recognition and obtaining appropriate imaging and cultures. Although outside the domain of emergency medicine, deep SSI treatment often requires aggressive surgical debridement. Empiric antibiotic treatment should be broad (eg, vancomycin or linezolid plus piperacillin-tazobactam or a carbapenem, or plus ceftriaxone and metronidazole).^43^ The primary surgical team should be consulted, particularly when prosthesis infection is suspected. As culture-directed therapy should be initiated as soon as microbiological analysis is available, early procurement of tissue, wound, and/or blood culture can aid in later antibiotic regimen honing.^43^

#### Surgical Site Collections

Swelling and tissue edema is normal and anticipated after most cosmetic surgeries. Such findings typically resolve after 1–2 months. However, persistent, organized collections may represent hematoma development.^58^ Hematoma occurrence varies depending on the procedure performed and the patient population, ranging from 3% to 15% in lipoabdominoplasty,^32^,^58^ and 0.6% to 5.7% in breast augmentation surgery.^62^–^65^ Risk factors for postoperative hematoma formation include anticoagulant use, older age, male gender, tobacco use, and medical comorbidities such as hypertension or malignancy.^66^–^68^ Hematomas usually occur in the initial 24-hour postoperative period but have been reported months following the initial procedure.^61^,^69^ Clinical presentation of hematomas depends on volume and rate of accumulation. Small hematomas are typically asymptomatic. More sizable hematomas with swelling, localized pain, and ecchymosis can typically be managed supportively.^61^ While rare, large hematomas with active bleeding can lead to hemodynamic instability and hemorrhagic shock, necessitating resuscitation and surgical intervention.^61^ Hematoma formation in patients with implanted prosthesis is a surgical emergency and should warrant close consultation with the surgical team for evacuation.

Implant rupture, especially in patients with breast augmentation, is an important cause of local fluid collections. The most common cause of implant rupture is age-related weakening of implant material.^70^ Signs and symptoms of implant rupture include contour deformity, volume diminution, palpable mass-like lesions, pain, and focal inflammation.^71^ Diagnosis of breast implant rupture on physical examination is feasible when presenting with typical features. However, clinical evaluation may fail to detect breast implant rupture that occurs over time without loss of breast volume and contour changes. Ultrasound and mammography are not sufficiently sensitive to rule out intracapsular ruptures, particularly of silicone implants.^72^ CT imaging has low sensitivity and is not recommended for evaluation of implant rupture.^73^ When feasible, magnetic resonance imaging (MRI) is the preferred study, but this is not required emergently. Sensitivities of clinical diagnosis, ultrasound, and MRI for implant rupture are 42%, 50%, and 83%, respectively, while specificities approach 50%, 90%, and 90%, respectively.^74^ Implant rupture is frequently asymptomatic and can be evaluated by MRI on an outpatient basis with surgeon follow-up.

In the subset of patients presenting with silicone injection-based cosmetic buttock enhancement, special attention must be paid to local collections, as foreign material is present in affected tissue. In addition to hematomas and seromas, these patients may have a foreign body reaction with granuloma formation.^26^ Most patients with this complication present with erythema, induration, and plaques (well-circumscribed, elevated, superficial, solid lesions) in the buttocks.^75^ Granulomatous reactions to silicone may occur months to years after silicone injection.^25^,^76^ Treatment of silicone granulomas can be challenging. Treatment modalities described in the literature include tetracyclines, steroids, and surgical excision.^25^,^77^

ED management consists of appropriate laboratory investigations to evaluate for blood loss and infection and imaging to evaluate collection size. In patients presenting with acute pain, other causes of abdominal discomfort should be considered before making a presumptive diagnosis of seroma or hematoma formation.^78^ Consultation with the surgical team is recommended to decide whether surgical drainage, needle aspiration, or close outpatient follow-up is appropriate. In hemodynamically unstable patients with evidence of hematoma, further investigation via ultrasound or CT angiography is necessary to search for bleeding sources including intraperitoneal foci.^78^,^79^

#### Postoperative Hemorrhage

Contemporary approaches to plastic surgery techniques have resulted in a less than 2% rate of postoperative bleeding.^80^ However, postoperative hemorrhage is associated with morbidity and mortality, accounting for roughly 4.5% of postoperative deaths in this population.^81^ Quantifying blood loss during cosmetic surgeries such as liposuction is difficult due to the composition of aspirate. However, it is estimated that for every 100 milliliters (mL) of aspirate, the average total body blood loss is 37 mL for females and 23 mL for males when not using tumescent solution, and an average of 0.5 to1.5 mL blood per 100 mL when tumescent technique is used.^82^ Most postoperative bleeding from cosmetic surgery is a result of capillary disruption, but cases of organ or vascular perforation with intraperitoneal hemorrhage have been reported.^83^ This hemorrhage can be further exacerbated by postoperative coagulopathy, including disseminated intravascular coagulopathy (DIC) secondary to a combination of hemodilution, hypothermia, and liposuction trauma.^58^ ED management consists of appropriate laboratory investigations to evaluate for blood loss and coagulation, as well as imaging assessment for hemorrhage via ultrasound or CT angiography.^84^ Hemodynamic resuscitation is a priority in the unstable patient.

#### Skin Necrosis and Wound Dehiscence

Flap compromise in the postoperative period is typically due to insufficient tissue perfusion secondary to disruption of subcutaneous perforating vessels and subdermal plexus. Flap compromise can lead to a variety of acute complications depending on depth of tissue involvement. Epidermolysis is the mildest variant in which only the epidermis suffers ischemia. The natural course of uncomplicated epidermolysis is spontaneous reepithelization without intervention.^61^ However, skin necrosis extending to subdermal tissue may involve severe pain and delayed healing. The incidence of skin necrosis varies between 3–4.4%, but less than 1% of these patients require revision.^32^ In most cases, necrosis leads to healing by secondary intention, which may require months to heal depending on the affected area size. Clinical features of skin necrosis include tenderness to palpation, ecchymosis, and tissue breakdown.^61^ Once detected, treatments include surgical debridement, antibiotics, and/or hyperbaric oxygen therapy.^37^

Wound dehiscence is a rare but important complication of plastic surgery, occurring in approximately 0.75% of patients.^85^ Wound dehiscence may occur secondary to infection, local collection, or necrosis. Risk of necrosis is heightened in procedures using autologous fat transfer, in which transplanted fat can cause localized inflammation and destruction of recipient tissues.^86^ ED management focuses on pain management and evaluation of any other underlying etiologies, most notably postoperative infection. Close follow-up with the primary surgeon is essential for wound debridement, dressing, and closure.

#### Venous Thromboembolism (VTE)

VTE is the leading cause of postoperative mortality in cosmetic surgery, accounting for up to 21% of postoperative deaths.^10^ Deep vein thrombosis (DVT) and pulmonary embolism (PE) incidence in liposuction is reported at less than 1%, but there is a marked increase in DVT incidence when liposuction is combined with other surgeries, especially abdominoplasty.^32^,^38^,^87^ Abdominoplasty has the highest incidences of DVT and PE in cosmetic surgery, up to 0.8% and 1.3%, respectively.^32^,^38^,^87^ These patients are more likely to experience long duration of surgery, impaired drainage of deep veins of the legs and pelvic area due to flexion at the hip during and after surgery, and higher incidence of postoperative inactivity.^88^ Risk of VTE increases significantly when cosmetic procedures are combined.^89^ There are no differences in imaging or treatment of VTEs in cosmetic surgery patients compared with other patient populations with suspected VTE.

#### Fat Embolism Syndrome (FES)

It is hypothesized that all patients undergoing liposuction surgery experience some degree of thromboembolic shower due to fat particles being dislodged during surgery, which can result in pulmonary fat embolism syndrome (FES).^90^ The underlying pathophysiology involves fat droplets from liposuctioned areas embolizing to the pulmonary circulation. Clinically significant FES carries an overall mortality rate of 10–15% and remains an important complication of cosmetic surgeries, especially SAL.^91^ FES is a multisystem disorder; primary clinical manifestations include tachycardia, respiratory distress, focal neurologic symptoms, and petechial rash.^92^ Respiratory dysfunction occurs frequently with severity varying from mild dyspnea and/or tachypnea to severe symptoms indistinguishable from acute respiratory distress syndrome.^92^ Neurologic manifestations occur in up to 80% of patients with FES and usually precede development of respiratory symptoms by 6–12 hours.^92^ Neurologic symptoms range from mild disorientation to coma.^93^ Petechiae on the upper body, primarily the head, neck, anterior chest, subconjunctiva, and axilla, are found in approximately 50% of FES patients.^91^ Petechial rash, which usually appears within three days of symptom onset, is believed to be the only pathognomonic feature of FES, However, the absence of a petechial rash should not exclude FES.^91^

Several approaches are suggested for FES diagnosis.^92^ CT is not useful for identifying the majority of fat emboli.^94^ Ventilation-perfusion scanning detects areas of perfusion mismatch, but cannot differentiate between VTE and FES.^95^ MRI is the most sensitive technique for demonstrating diffuse ischemic cerebral changes of FES.^93^,^96^–^98^ In the acute setting, FES diagnosis is clinical, with imaging as an adjunct to eliminate alternative diagnoses.^92^ Treatment considerations include maintenance of fluid and electrolyte balance, administration of supplemental oxygen, and endotracheal intubation with mechanical ventilator support when required.^93^ Anticoagulation is not recommended, as fat emboli are a distinct clinical entity from thromboembolism and not amenable to thrombolysis.^93^

#### Visceral Perforation

Visceral perforation is an important complication requiring aggressive intervention. As cosmetic surgery is routinely performed in an ambulatory setting, patients may not be evaluated by their surgeon until three or four days postoperatively. Therefore, these patients may present to the ED for evaluation.^48^,^99^,^100^ Bowel wall perforation with visceral injury is the second most common cause of mortality after liposuction, with an incidence of 14 per 100,000 procedures.^101^,^102^ Ileal perforation is most common, followed by perforation of the jejunum, spleen, cecum, and transverse and sigmoid colon.^100^ Risks for perforated viscus during liposuction include morbid obesity, previous surgical scars, divarication of recti, and abdominal wall hernias.^58^ Patients may present subtly, with pain out of proportion to postoperative course, or in shock.^78^ Perforation may extend to surrounding lymphatic, vascular, and intra-abdominal structures, or may occur far from the original surgical site, as in the case of patients with severe chest pain and dyspnea, possibly indicating perforation into the thorax.^78^,^103^

In the ED, patients with severe abdominal pain after cosmetic surgery should be assessed carefully for visceral perforation. While diagnosis of peritonitis is primarily clinical, plain radiographs of the abdomen or chest in upright position and CT may be useful adjuncts in confirming diagnosis.^100^,^103^ Management of severe peritonitis is complex and requires a multidisciplinary approach consisting of surgical evaluation and aggressive resuscitation with hemodynamic support, broad spectrum antibiotics, and IV fluids.^104^

#### Local Anesthetic Systemic Toxicity (LAST)

LAST is a potentially devastating complication of local anesthesia administration. The United States Food and Drug Administration recommends a maximum dose of 7 milligrams per kilogram (mg/kg) of lidocaine for local anesthesia.^105^ However, when used during tumescent liposuction, this ceiling increases to 35–65 mg/kg.^105^,^107^ This has proven acceptable, as plasma concentrations of lidocaine remain at subtoxic levels despite high infiltrative dosages, affirming that tumescent lidocaine is absorbed slowly from subcutaneous tissues producing lower peak blood levels vs other administration routes.^108^ Up to 30% of the anesthetic is suctioned after infiltration, decreasing systemic absorption.^109^,^110^

Serum lidocaine concentrations peak between 12–16 hours following tumescent infiltration, presumably when the patient is home following office-based procedures.^106^,^111^ Various concentrations of epinephrine are described, typically between 0.65 mg/Liter (L) and 1 mg/L. Maximal doses do not exceed 7 mg/kg.^106^,^111^ Epinephrine use may increase post-SAL cardiac index, delaying potential LAST-associated cardiovascular collapse. Typical tumescent solution lidocaine concentration is one gram (g) per bag, containing 1110 mL or 0.9 g/L (0.09% lidocaine).^108^ Sodium bicarbonate is added to reduce the discomfort of large-volume subcutaneous, tumescent infiltration.^108^

**Figure 3:**
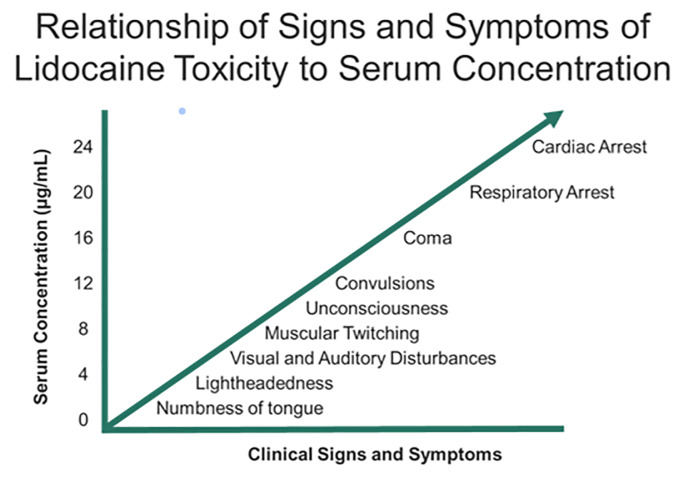
Relationship of signs and symptoms of lidocaine toxicity to serum concentration.

Systemic complications of tumescent anesthesia may result from an allergic response or medication toxicity from epinephrine or local anesthetic. Allergic reactions with urticaria, angioedema, and/or anaphylaxis should be treated with antihistamines, intramuscular/IV epinephrine, and airway support as necessary. Medication toxicity may result from direct infiltration into large vessels or impaired drug metabolism (hepatic dysfunction or pseudocholinesterase deficiency for local anesthetics).^112^ LAST presentation is variable. Toxicity involves a continuum of adverse central nervous system effects progressing to cardiovascular symptoms at increasing dosages (Figure 3).^112^ Typical prodromal symptoms (eg, circumoral numbness, metallic taste, auditory changes) occur in approximately 18% of patients, although these are decreased in the presence of general anesthesia.^113^ In fulminant presentations, these patients may present with seizures and cardiovascular collapse.

**Figure 4:**
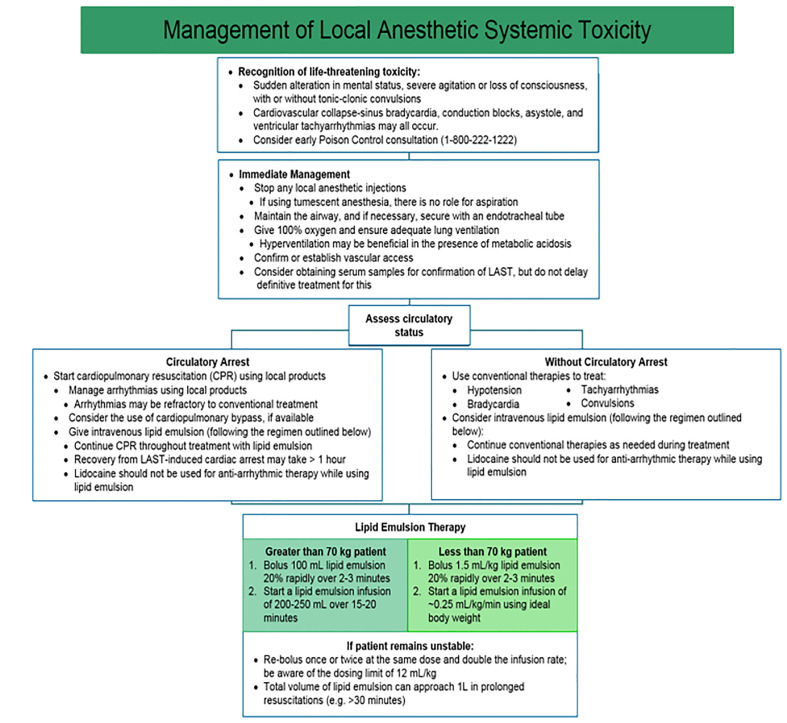
Evaluation and treatment algorithm for local anesthetic systemic toxicity.

The American Society of Regional Anesthesia and Pain Medicine stresses the unique circumstances of resuscitation in patients with LAST (Figure 4).^113^ In the peri-arrest period, aggressive airway management to prevent hypoxia and acidosis may slow seizures and cardiovascular collapse. Seizures are managed primarily with benzodiazepines and lipid emulsion therapy.^114^ Current lipid emulsion therapy recommendations call for bolus injection of 1.5 mL/kg IV followed by an infusion at 0.25 mL/kg/min.^114^ Beyond standard life support measures, providers managing cardiac arrest secondary to LAST should consider amiodarone for ventricular arrhythmias, as further lidocaine use may worsen toxicity. Negative inotropic agents are contraindicated, as they may precipitate or worsen myocardial depression.

## LIMITATIONS

This is a narrative review, and thus no pooling of data from individual studies was conducted. We did not assess article quality or risk of bias. Much of the included literature consists of studies conducted in non-emergent settings, and thus generalizing these studies to the ED setting is challenging. Much of the information and resources come from society guidelines.

## CONCLUSIONS

As a result of the increasing number of cosmetic surgeries performed, rising cosmetic tourism, and lack of legal restrictions on who may perform these procedures, post-cosmetic surgery patients may present to the ED with a variety of complications. The most common issues include postoperative wound collections and infections, VTE, hemorrhage, and medication toxicity. These complications are associated with severe morbidity if diagnosis is delayed. Other significant complications include syncope, skin necrosis, and intra-abdominal injury. Critical patients should be evaluated in the resuscitation bay, and consultation with the primary surgical team is essential. Understanding these complications and their management is essential to minimizing the morbidity and mortality accompanying these cosmetic surgical procedures.
